# Effects of organic matter and low oxygen on the mycobenthos in a coastal lagoon

**DOI:** 10.1111/1462-2920.14469

**Published:** 2018-12-13

**Authors:** Ana‐Sofia Ortega‐Arbulú, Monica Pichler, Aurèle Vuillemin, William D. Orsi

**Affiliations:** ^1^ Department of Earth and Environmental Sciences, Paleontology and Geobiology Ludwig‐Maximilians‐Universität München 80333 Munich Germany; ^2^ GeoBio‐Center Ludwig‐Maximilians‐Universität München 80333 Munich Germany

## Abstract

Fungi living in sediments (‘mycobenthos’) are hypothesized to play a role in the degradation of organic matter deposited at the land‐sea interface, but the environmental factors influencing the mycobenthos are poorly understood. We used mock community calibrated Illumina sequencing to show that the mycobenthos community structure in a coastal lagoon was significantly changed after exposure to a lignocellulose extract and subsequent development of benthic anoxia over a relatively short (10 h) incubation. Saprotrophic taxa dominated and were selected for under benthic anoxia, specifically *Aquamyces* (Chytridiomycota) and *Orbilia* (Ascomycota), implicating these genera as important benthic saprotrophs. Protein encoding genes involved in energy and biomass production from Fungi and the fungal‐analogue group Labyrinthulomycetes had the highest increase in expression with the added organic matter compared with all other groups, indicating that lignocellulose stimulates metabolic activity in the mycobenthos. Flavobacteria dominated the active bacterial community that grew rapidly with the lignocellulose extract and crashed sharply upon O_2_ depletion. Our findings indicate that the diversity, activity and trophic potential of the mycobenthos changes rapidly in response to organic matter and decreasing O_2_ concentrations, which together with heterotrophic Flavobacteria, undergo ‘boom and bust’ dynamics during lignocellulose degradation in estuarine ecosystems.

## Introduction

Mycoplankton and the mycobenthos are groups of aquatic Fungi that feed via osmotrophy in planktonic and sedimentary settings respectively (Richards *et al*., 2012). Fungi in freshwater ecosystems are stimulated by the availability of organic matter, particularly plant‐derived detritus such as leaves, which plays an important first step in the degradation of terrestrial organic matter (Gessner and Schwoerbel, 1991; Nikolcheva *et al*., 2003; Grossart and Rojas‐Jimenez, 2016; Fabian *et al*., 2017). The mycobenthos are also ubiquitous in shallow water marine sediments in coastal ecosystems (Newell *et al*., 1989; Newell, 1994; Gessner *et al*., 1997; Mohamed and Martiny, 2011; Dini‐Andreote *et al*., 2016). However, it is poorly understood how the mycobenthos react to changing environmental conditions in estuarine habitats, where there is an annual flux of organic matter amounting to ~0.5 Pg C (Hedges *et al*., 1997; Benner, 2004).

Estuarine ecosystems characterized by dense vegetation are large sources of organic matter (e.g. salt marshes, seagrass meadows and mangroves), and are considered ‘hidden forests’, storing a similar amount of carbon as the rainforest and thus play a critical role in Earth's climate (McLeod *et al*., 2011). Benthic microbial communities living in these coastal ecosystems drive organic matter decomposition, nutrient regeneration and influence water column dissolved O_2_ concentrations (Nixon, 1981; Boynton and Kemp, 1985). Given the importance of Fungi in degrading organic matter in terrestrial ecosystems (Tedersoo *et al*., 2014; Treseder and Lennon, 2015), the mycobenthos could potentially play a similar role in the degradation and remineralization of organic matter deposited in estuarine ecosystems.

Fungi in coastal habitats have been found previously to associate with, and degrade plant material (Fell and Master, 1980; Newell *et al*., 1989; Newell, 1994; Raghukumar, 2004). The resulting production of fungal biomass can even serve as a food source for larger animal predators such as snails (Silliman and Newell, 2003). Fungi can represent a major component of the initial microbial community that colonizes freshly bioturbated sediments (Taylor and Cunliffe, 2015) – indicating that they are potentially important for seafloor carbon turnover processes. Further evidence for a role of Fungi in estuarine organic matter turnover has been demonstrated by ergosterol‐based methods showing that fungi grow on *Spartina* leaves in salt marshes (Newell *et al*., 1989; Newell, 1994) and stable isotope probing experiments has shown that mycobenthos utilize lignocellulose as a carbon source in estuarine sediments (Gontikaki *et al*., 2015). In salt marshes the mycobenthos can account for up to 10% of total detrital biomass indicating they are important members of the ecosystem (Gessner *et al*., 1997). Fungi are also more abundant and diverse in the marine benthos with increasing proximity to the coast (Burgaud *et al*., 2013; Barone *et al*., 2018), reflecting their positive selection in coastal ecosystems including estuaries (Richards *et al*., 2012).

However, the environmental factors influencing the turnover and community structure of the mycobenthos in coastal ecosystems are still poorly understood. Given the preference of many Fungi to utilize plant‐derived organic matter in nutrient rich terrestrial environments (Gessner *et al*., 1997; Treseder and Lennon, 2015), it has been hypothesized that the growth of the marine mycobenthos is stimulated by inputs of organic matter (Richards *et al*., 2012). To test this hypothesis, and constrain the parameters influencing the mycobenthos in shallow water coastal sediments, we performed a replicated controlled experiment with sediments from Sage Lot pond, a coastal lagoon that represents a subestuary of the Waquoit Bay on Cape Cod (Massachusetts, USA).

We applied a lignocellulose extract as a proxy for plant‐derived organic matter that can be deposited in large abundance in coastal lagoons containing salt marshes and seagrass meadows (McLeod *et al*., 2011). Oxygen was measured throughout the incubation and samples were taken to measure mycobenthos community structure via high‐throughput sequencing of internal transcribed spacer (ITS1) regions at varying oxygen concentrations (84%, 25%, 5% and 0% atmospheric oxygen saturation). Metatranscriptomes were also performed under 25% atmospheric oxygen saturation to assess transcriptional activity of the total microbial community, and bacterial abundance, community structure and gene expression was also assessed. The results show that community structure and potential trophic status of the mycobenthos can be significantly altered by organic matter and low O_2_ conditions, and that complex microbial consortia consisting of bacteria and mycobenthos work together to break down lignocellulose in the estuarine sediments. To our knowledge, these are the first experimental data to characterize a response of the mycobenthos to changing organic matter inputs and O_2_ concentrations in an estuarine habitat.

## Results

### *Mock community analysis*

**Figure 1:**
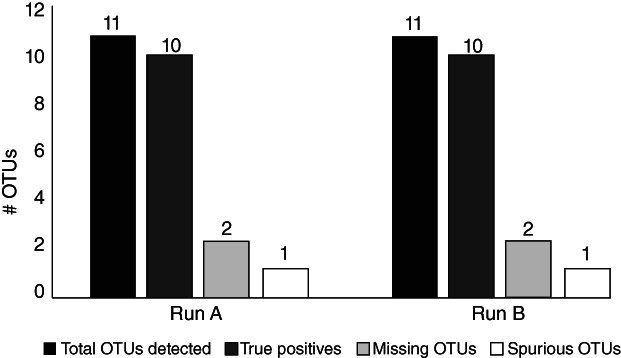
Results of mock community ITS1 sequencing across two separate sequencing runs.

Two separate ITS1 sequencing runs of ‘mock communities’ containing 13 different species of Fungi (Table 1) were sequenced alongside the environmental samples as a quality control procedure. This allowed us to validate that the clustering threshold used for ITS1 sequences from the environmental samples (97% similarity), produced true species‐level operational taxonomic units (OTUs). The analysis showed that at 97% sequence identity there were 11 total OTUs, 1 spurious OTU and 10 true positives out of 13 total species (Fig. 1). While the exact number of OTUs in the mock community was not obtained, mock communities are rarely recovered at the exact richness after high‐throughput sequencing with variability reaching >30% of the original community even under stringent criteria (Edgar, 2013). We also sequenced negative controls (dust samples from the lab and DNA extraction blanks) (Supporting Information Table S1) and removed any OTUs that overlapped with the experimental samples (<10%). Thus, we interpret the remaining fungal OTUs deriving from ITS1 sequences from our environmental samples to be representative of roughly species‐level taxa that are endemic to our samples and not the result of contamination.

**Table 1:** Composition of the fungal mock community and sequencing depth of ITS1 regions from each strain.

			Number of reads	
Fungal strain in mock community	DSM #	Isolation source	Run A	Run B
*Aspergillus oryzae*	1862	Food fermentations	164 658	4803
*Candida mesenterica*	70 759	Beer	92 130	59 563
*Nakazawaea holstii*	70 764	Fluid coffee extract	49 519	16 086
*Endomyces fibuliger*	70 554	Forest soil	24 794	8098
*Metschnikowia pulcherrima*	27 438	Floral nectar	73 845	31 431
*Pichia fermentans*	70 090	Brewer's yeast	77 349	34 032
*Pichia norvegensis*	70 760	Margarine	27 523	12 102
*Rhodotorula bogoriensis*	70 872	*Randia malleifera*, Leaf	29 095	10 825
*Millerozyma farinosa*	2226	Fermenting cacao	9573	3956
*Kluyveromyces marxianus*	5418	Meadowgold creamery	6138	3384
*Lipomyces tetrasporus*	70 314	Soil	12	8
*Lipomyces starkeyi*	70 295	Soil	3	3
*Dipodascus magnusii*	3189	Mucilaginous secretion of oak	–	–

### *O_2_ consumption*

**Figure 2:**
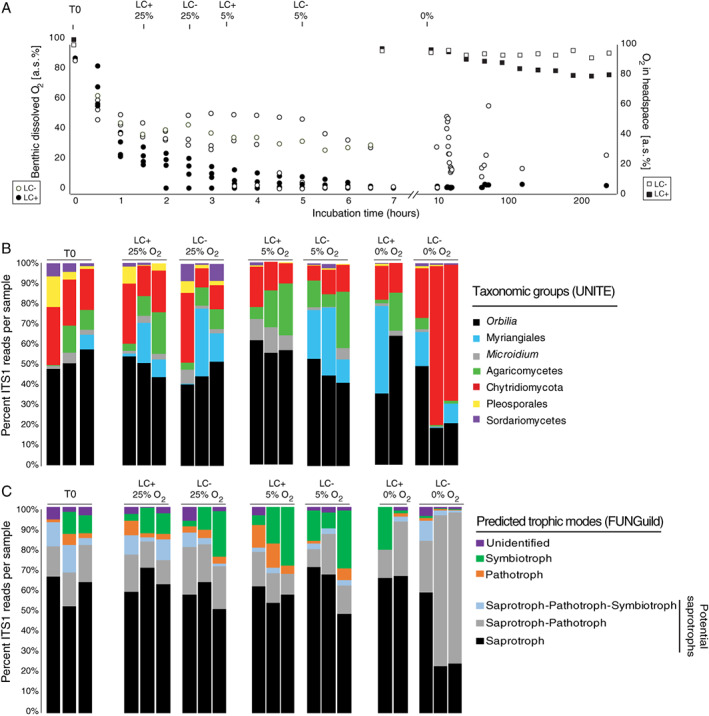
A. Dissolved oxygen consumption over time at the benthic interface (circles) and gas in the headspace (squares) in incubations with (LC+, filled circles) and without (LC‐, clear circles) lignocellulose additions. Vertical lines represent times for sampling for DNA extraction. B. Relative abundance of the fungal taxa in biological replicates sampled at varying oxygen concentrations in samples that did, or did not, receive lignocellulose. LC+: lignocellulose addition, LC‐: control. C. Predicted trophic modes of the same fungal taxa in the biological replicates shown in panel B using FUNGuild (Nguyen *et al*., 2016).

O_2_ was consumed faster at the sediment seawater interface in incubations treated with lignocellulose than in controls, indicating that lignocellulose stimulated benthic microbial respiration and activity (Fig. 2A). While benthic O_2_ was consumed to levels below detection at the sediment surface in all replicates after 7 h, O_2_ in the headspace was >75% atmospheric saturation (a.s.) throughout the incubations. Headspace O_2_ in the presence of lignocellulose was depleted faster relative to the control (Fig. 2A). The data indicate that while aerobic conditions existed in the flasks throughout the incubation, increased benthic microbial respiration in the presence of lignocellulose created a zone of anoxia at the sediment seawater interface after 7 h. Because O_2_ concentrations are tightly coupled with microbial physiology and metabolic activity, we selected sampling time points for ITS1 sequencing and metatranscriptomics based on specific benthic O_2_ concentrations.

### *Changes in mycobenthos community structure*

**Figure 3:**
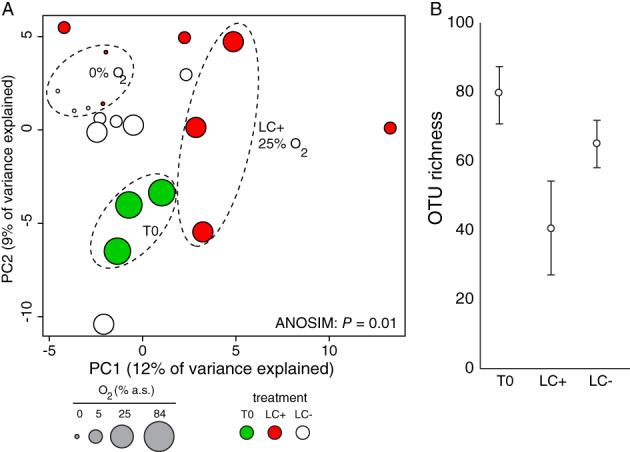
A. Principal Component Analysis of ITS1 OTU relative abundance. Note, the combined effects of lignocellulose and oxygen concentration on mycoplankton diversity and abundance are statistically significant. Circles are drawn around biological replicates sharing similar communities that occupy distinct space in the ordination. B. Richness of ITS1 OTUs recovered at T0, and also with and without lignocellulose extract. Error bars represent standard deviations.

A total of 146 891 sequences were obtained from Illumina sequencing of ITS1 amplicons from the incubations, which corresponded to 369 OTUs that passed our quality control procedures. The starting community was dominated by ITS1 sequences affiliated with the ascomycete genus *Orbilia*, followed by OTUs affiliated with the Chytridiomycota, Sordariomycetes, Pleosporales and Agaricomycetes (Fig. 2B). After establishment of benthic anoxia, samples receiving the lignocellulose extract were dominated by OTUs affiliated with *Orbilia* whereas control incubations were dominated by Chytridiomycota (Fig. 2B). The combined effects of lignocellulose and benthic O_2_ concentration had a statistically significant effect on diversity in the mycobenthos (ANOSIM: *P* = 0.01) (Fig. 3A).

**Figure 4:**
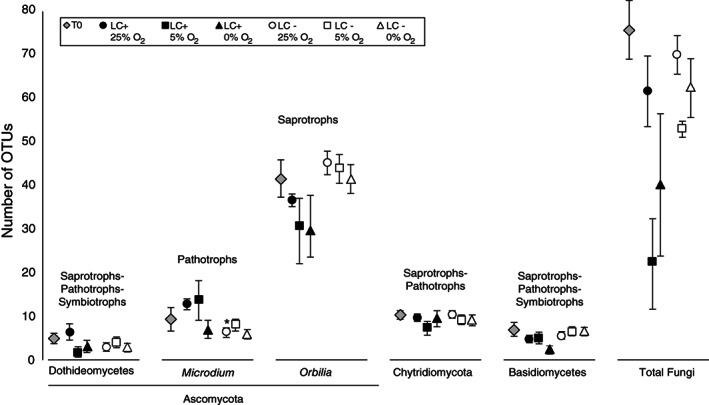
Richness of fungal groups across the different experimental timepoints and treatments. Predicted trophic modes with FUNGuild (Nguyen *et al*., 2016) are shown. Error bars represent standard deviations across three biological replicates.

**Figure 5:**
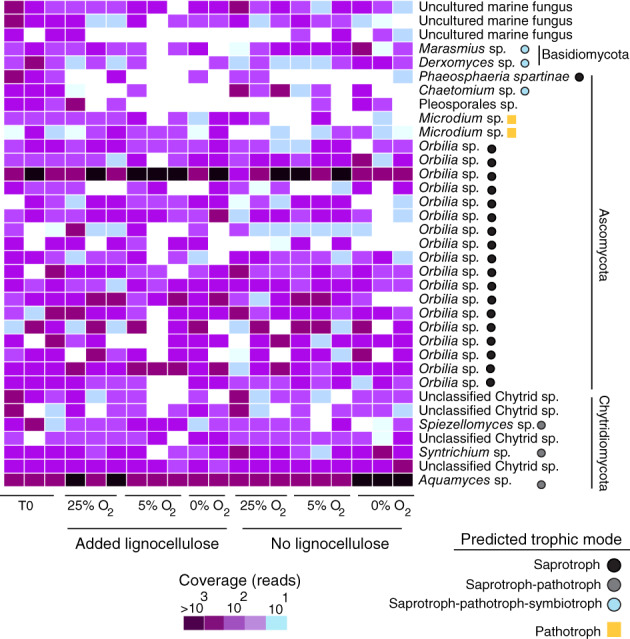
Changes in relative abundance of the most abundant OTUs in the mycobenthos over the experiment, with predicted trophic modes indicated for groups where a genus level annotation is possible.

The highest richness was observed in the ascomycete genus *Orbilia,* compared with the other mycobenthos taxa present throughout the incubations (Fig. 4). When benthic O_2_ reached 25% a.s., samples receiving the lignocellulose extract exhibited increases in OTUs affiliated with *Microdium* whereas the number of OTUs affiliated with *Orbilia* decreased (Fig. 4). After benthic O_2_ levels dropped to 5% a.s., the richness of the mycobenthos decreased relative to oxic conditions in the presence of added organic matter (Fig. 4). Upon development of benthic anoxia, there was a decrease in the richness of OTUs affiliated with Basidiomycetes in the presence of lignocellulose (Fig. 4). The most abundant taxon was affiliated with the Chytrid genus *Aquamyces* (reads = 35 332; 24% of total sequences) and the second most abundant taxon was affiliated with the ascomycete genus *Orbilia* (reads = 15 519; 11% of total sequences)(Fig. 5).

### *Predicted trophic modes*

We used FUNGuild (Nguyen *et al*., 2016) to predict the trophic mode of OTUs in the mycobenthos, which was able to assign a putative trophic lifestyle to >90% of OTUs detected in each sample (Fig. 2C). This analysis showed that OTUs affiliated with pathotrophic, symbiotrophic and saprotrophic taxa were present in the sediment at the beginning of the incubation (Fig. 2C). After development of benthic anoxia, OTUs affiliated with saprotrophic taxa dominated the mycobenthos both in the presence and absence of the lignocellulose extract, whereas OTUs affiliated with pathotrophic and symbiotrophic taxa exhibited lower abundance relative to T0 (Fig. 2C). After the development of benthic anoxia, the most abundant OTUs were affiliated with the saprotrophic genera *Aquamyces* and *Orbilia* (Fig. 5). These findings implicate both *Aquamyces* and *Orbilia* as saprotrophic degraders of organic matter in the estuarine benthos, also under anoxic conditions.

### *Metatranscriptomics*

**Figure 6:**
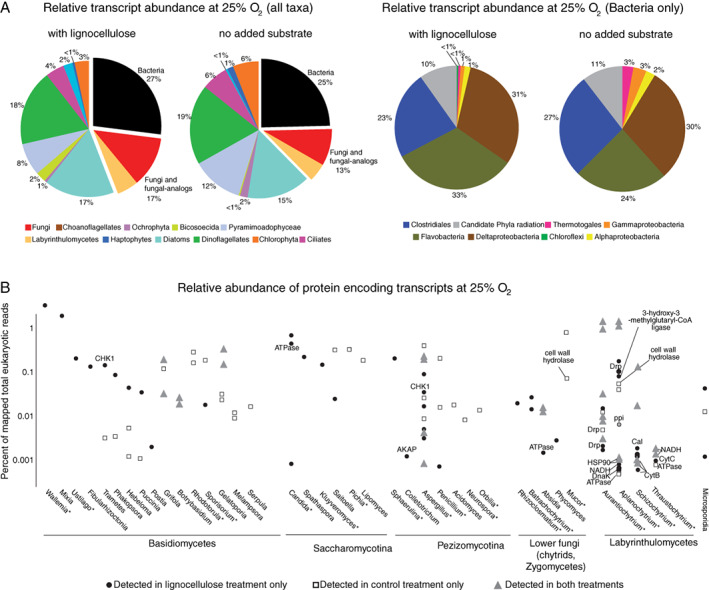
A. Taxonomic distribution of expressed genes recovered in the metatranscriptomes. The pie charts on the right showing individual bacterial taxa are derived from the black sections of the pie charts on the left. B. Expressed genes from fungi and fungal‐analogues in metatranscriptomes from the benthic 25% O_2_ a.s. timepoint. All non‐labelled genes encode hypothetical proteins. Asterisks mark genera that are typically encountered in marine samples (Richards *et al*., 2012). Gene abbreviations are as follows. CHK1: checkpoint‐1 protein kinase (signal transduction), AKAP: A‐anchor protein kinase (signal transduction), ppi: peptidyl‐prolyl cis‐transisomerase (protein folding), Drp: dehydration resistance protein, ATPase: ATP synthase (energy production), CytB: cytochome b (energy production), CytC: cytochrome C (energy production), NADH: NADH dehydrogenase (energy production), Cal: Calmudulin (signal transduction), DnaK: (DNA replication).

Genes encoding proteins expressed by microbial eukaryotes dominated the metatranscriptomes in both the lignocellulose (average coverage = 358 554 reads; 73% total mapped reads) and control treatments (average coverage = 433 056 reads; 75% total mapped reads) at 25% O_2_ a.s. (Fig. 6A). Of those expressed genes assigned to microbial eukaryotes, 17% were assigned to fungi and fungal‐analogue taxa in the lignocellulose treatment and 13% in the control treatment. The increased amount of relative gene expression (4% increase) in the presence of lignocellulose extract from the fungi and fungal‐analogues was higher than any other group of microbial eukaryotes, and also higher than bacteria (which decreased 2%) (Fig. 6A). The fungal genus *Aspergillus*, as well as the saprotrophic Labyrinthulid genera *Aurantiochytrium*, *Thraustochytrium*, *Alpanochytrium*, *Schizochytrium* displayed the highest number of expressed genes, including those encoding energy producing cytochromes, NADH‐dehydrogenases and ATPases (Fig. 6B). High relative expression genes encoding hypothetical proteins from the basidiomycete genera *Wallemia*, the ‘brown rot’ fungus *Postia*, and the ‘white rot’ fungus *Trametes* (Vasina *et al*., 2017) were detected (Fig. 6B). None of these taxa were identified as contaminants in the sequencing controls, and thus their presence and activity in the marine sediments are interpreted to be authentic.

The expressed genes from Fungi and fungal‐analogues encoding CHK1, calmodulin and DnaK proteins (Fig. 6B) are indicative of signal transduction pathways and an active cell cycle (Tatusov *et al*., 1997), which indicates the mycobenthos contributed to the community respiration (Fig. 2A). Several genera within the Basidiomycota (*Wallemia*, *Ustilago*, *Mixia*) and Ascomycota (*Candida*, *Spathaspora*, *Kluyveromyces*) were only observed to express genes in the presence of lignocellulose, at relatively high levels compared with most other transcriptionally active groups (Fig. 6B). The ‘brown rot’ fungus *Postia* and the ‘white rot’ fungus *Trametes* (Vasina *et al*., 2017) also exhibited a relatively higher gene expression in the lignocellulose treatments – suggesting that their activity was stimulated by this substrate. Most of the genes expressed encode hypothetical proteins with as‐of‐yet no biochemically determined function (Fig. 6B).

### *Bacterial community structure and gene expression*

**Figure 7:**
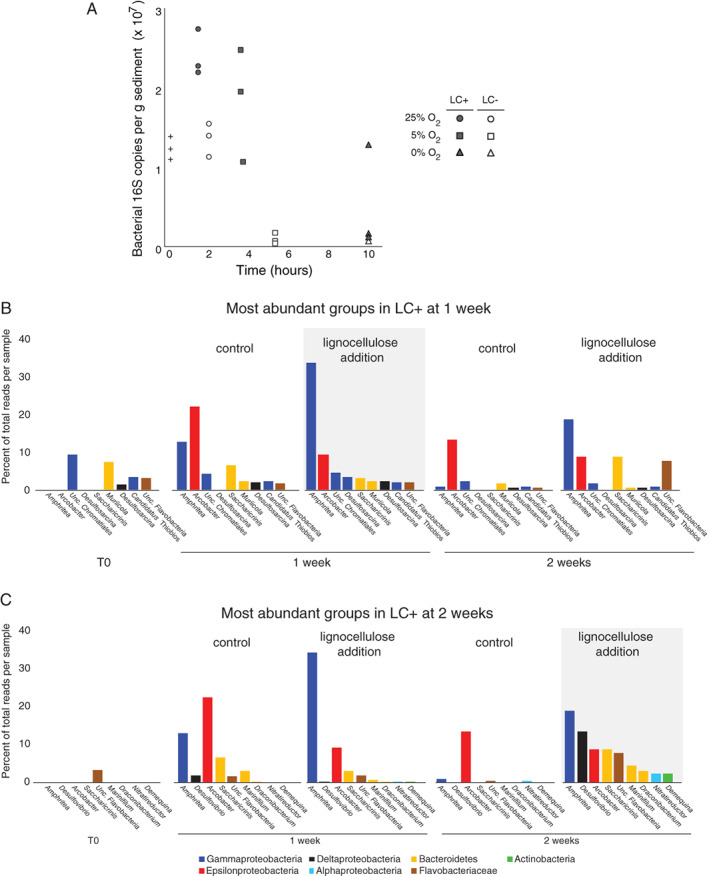
A. Bacterial qPCR of 16S rRNA genes at specific benthic oxygen concentrations with (filled shapes) and without (clear shapes) lignocellulose. T0 is represented by ‘+’ symbols. Relative abundance of the top 10 most abundant bacterial OTUs at 1 (panel A) and 2 (panel B) week incubation times (anoxic benthic conditions) with, or without, lignocellulose. LC+: lignocellulose addition, LC‐: control.

Addition of the lignocellulose extract stimulated the growth bacteria within the first 2 h, as measured via qPCR of 16S rRNA genes (Fig. 7A). However, after 10 h following the development of benthic anoxia, bacterial 16S rRNA genes decreased by over an order of magnitude indicating that their biomass was turned over relatively quickly (Fig. 7A). After 1 and 2 weeks of incubation with benthic anoxia and oxygenated headspace (Fig. 2A), increased abundances of Flavobacteria, denitrifying *Nitratireductor*, sulfate reducing bacteria related to *Desulfovibrio* and *Desulfosarcina,* and fermentative *Saccharicinis* (Bacteroidetes) were observed in the lignocellulose treatments, relative to the control (Fig. 7B and C). Metatranscriptomes sampled at 25% O_2_ a.s. showed that gene expression from bacteria was dominated by Clostridiales, Flavobacteria, Deltaproteobacteria and representatives of the Candidate Phyla Radiation – with expressed genes from Flavobacteria increasing 9% in the presence of lignocellulose extract relative to the control (Fig. 6A). Thus, in addition to the mycobenthos, the availability of organic matter and resulting anoxia had a marked effect on the turnover and assembly of benthic, anaerobic bacterial communities.

## Discussion

Our study shows the community structure of the mycobenthos can change rapidly, on the scale of hours, in response to increased availability of lignocellulose and low benthic O_2_ concentrations. Their relative quick response to the rapidly changing conditions in our experiment suggest that the active responding fungi, mostly *Aquamyces* and *Orbilia,* may correspond to ecological r‐strategists in estuarine sediments. A ‘boom and bust’ (Lennon and Jones, 2011) type response of mycobenthos populations would explain why the communities change quickly in response to the added organic matter, and then again a few hours later after the O_2_ is depleted (Fig. 3A). The speed at which we observed change in the mycobenthos communities is much faster in comparison to mycoplankton communities that change over longer timescales during phytoplankton blooms (Gutierrez *et al*., 2011; Taylor and Cunliffe, 2016).

The fastest growing eukaryotic microbe (a thermophilic yeast) was observed to have a doubling time of 52 min (Groeneveld *et al*., 2009). Thus, it is highly unlikely that the changes in the community observed over the course of 2–4 h were due solely to increases in cellular division rates in the mycobenthos. Since numerous forms of organic matter (e.g. acetate, propionate, alcohols, trehalose,and yeast extract) can serve as chemical activators of fungal spore germination (Emerson, 1948; Cotter, 1981; Rivero and Olmedo‐Cerda, 1987), it is more likely that dissolved organic matter resulting from enzymatic digestion of the added lignocellulose extract activated the germination of fungal spores or dormant cells in the mycobenthos. Fungal spores have been found to be activated within 1 h after exposure to acetate and propionate (Rivero and Olmedo‐Cerda, 1987), which is on the timescales of changing community structure seen in response to organic matter in the sediments studied here.

However, activation of dormant fungal cells by the added organic matter could also explain the rapid changes in the mycobenthos community structure observed in our experiment. Similarly, bacterial communities in coastal sediments contain large numbers of dormant cells, whose activity fluctuates on hour timescales in response to rapid changes in O_2_ consumption due to increased nutrient additions (Kearns *et al*., 2016). Our finding that the number of mycobenthos taxa decreases under nutrient addition (Fig. 3B) is also similar to the effects of nutrient addition on Bacteria in salt marsh sediments, where increased nutrients causes a loss of diversity in the active fraction of the bacterial community (Kearns *et al*., 2016).

Several of the most abundant mycobenthos OTUs at T0 were present throughout the entire incubation (Fig. 5), indicating that these naturally abundant *in situ* taxa are capable of surviving over a range of benthic O_2_ concentrations. As sequencing of the mock community showed that our ITS1 sequencing and clustering approach recovers a realistic species richness (Fig. 1), defined here as <30% variability in the original mock community (Edgar, 2013), the mycobenthos OTUs should correspond to roughly species level taxa. The most abundant mycobenthos taxa at T0 consisted of OTUs affiliated with saprotrophic‐pathotrophic Chytridiomycota (*Aquamyces*, *Syntrichium*), the saprotrophic ascomycete genus *Orbilia* and multiple OTUs affiliated with the Basidiomycota and Ascomycota (Fig. 5).

A dominant OTU affiliated with *Aquamyces* also exhibited positive selection under anoxia, as its relative abundance increased under this condition (Fig. 5). *Aquamyces* has a global distribution in aquatic freshwater habitats, where it has been proposed to be an important saprotroph and degrader of plant‐derived organic matter (Letcher *et al*., 2008). *Aquamyces* species utilize relatively less reactive substrates including chitin, cellulose, keratin, lignin and sporopollenin in aquatic habitats (Shearer *et al*., 2007). This trait of Chytrid fungi might make them more successful in aquatic habitats that receive large amounts of terrestrial and plant derived (e.g. from macrophytes) organic matter. For example, in freshwater sediments Chytrid fungi can be the most dominant group of fungi (Freeman *et al*., 2009). Chytrid fungi are also ubiquitous in seawater from temperate to arctic latitudes (Richards *et al*., 2012; Comeau *et al*., 2016; Hassett and Gradinger, 2016; Taylor and Cunliffe, 2016; Hassett *et al*., 2017), and these global distributions, together with their saprotrophic trophic mode and capability of surviving over a range of oxygen concentrations, implicate them as potentially important degraders of organic matter in the benthos.

The dominance of OTUs affiliated with *Orbilia* in the sampled lagoon is consistent with their lifestyle as saprotrophic fungi that are usually found in rotten wood from tropical and temperature regions (Swe *et al*., 2009; Pearman *et al*., 2010). The diversity of *Orbilia* has been observed to decrease in estuarine habitats due to increasing salinity (Pearman *et al*., 2010), indicating a potential preference for estuarine conditions as opposed to truly marine habitats. To our knowledge, these is the first experimental data that implicate *Aquamyces* and *Orbilia* as dominant saprotrophs in a marine benthic ecosystem that can persist during anoxia. Benthic anoxia is a common feature of the sampled environment in Waquoit Bay (Cape Cod, USA), where increased nitrogen input to the watershed through atmospheric deposition, fertilizer and wastewater has lead to an increase in primary productivity and recurrent bottom water anoxia (Valiela *et al*., 1992; D'Avanzo and Kremer, 1994). An increased frequency of benthic anoxic events in the Waquoit Bay watershed are correlated with a change in benthic fauna (Valiela *et al*., 1992), which is consistent with benthic anoxia imparting a strong selective pressure on the mycobenthos in our incubations from the lagoonal sediments (Fig. 3A).

Because the relative increase in gene expression from fungi and fungal‐analogues was higher than any other microbial group (including bacteria) in the presence of  lignocellulose extract (Fig. 6A), addition of this substrate appears to have induced their metabolic activity. However, there was no significant difference in average relative expression of specific protein‐encoding genes expressed per group in the presence or absence of lignocellulose. This could be explained by their active utilization of lignocellulose, or lignocellulose derived substrates, in the natural environment (e.g. in the controls). The fungal genus *Aspergillus*, and saprotrophic Labyrinthulid genera *Aurantiochytrium*, *Thraustochytrium*, *Alpanochytrium*, *Schizochytrium* were the most transcriptionally active – as these groups expressed the highest number of genes relative to other groups in the mycobenthos (Fig. 6B).

While the PCR primers used for ITS1 sequencing do not detect the ‘fungal‐analogue’ groups Labyrinthulids and Thraustochytrids, an abundance of genes expressed by these groups in the metatranscriptomes shows that they were also important in the turnover of lignocellulose. For example, expressed genes encoding cell wall associated hydrolases from Labyrinthulids (Fig. 6B) indicate the production of secreted enzymes that act on organic matter. The heightened activity of Labyrinthulomycetes in response to lignocellulose might represent a unique feature of lignocellulose degradation in marine systems compared with terrestrial systems – where traits conferring lignocellulose degradation (lignin peroxidases, cellobiohydrolases, crystalline cellulases) are dominated by filamentous Fungi (Treseder and Lennon, 2015). Our findings showing an increase in activity from Labyrinthulomycetes in the presence of organic matter is consistent with the hypothesis that complex polysaccharides are a major nutritional source for planktonic Labyrinthulomycetes (Raghukumar and Damare, 2011) and extend this ecological role into the benthos.

The high relative expression of a hypothetical protein encoding gene from *Wallemia* in the presence of lignocellulose (Fig. 6B) is consistent with the adaptation of this genus to hypersaline environments, a trait that is rare in the Basidiomycota (Padamsee *et al*., 2012). *Wallemia* produces carbohydrate active enzymes (CAZymes) involved in the degradation of plant polysaccharides (Rytioja *et al*., 2014), which may have contributed to the utilization of the added lignocellulose over the course of the experiment. A role of fungal enzymes in degrading plant derived organic matter in marine habitats was demonstrated by expression of CAZyme‐encoding genes by fungi in marine sediments that target plant material (Orsi *et al*., 2018), and increased enzymatic activity of fungi in estuaries compared with offshore locations (Gutierrez *et al*., 2017). We did not detect *Wallemia* in the ITS sequences, which could be due to PCR primer biases or a low abundance of *Wallemia* cells that have a relatively high transcriptional activity per cell compared with other members of the mycobenthos. Conversely, *Aquamyces* and *Orbilia* were not detected in the metatranscriptomes despite being abundant in the ITS datasets. However, this is due to a database bias in since to our knowledge there are no genomes yet available from representatives of *Aquamyces* and *Orbilia*. This discrepancy between the ITS and metatranscriptome data shown in our study highlights the need for sequenced genomes from ecologically relevant aquatic fungi (for example from the Chytridiomycota), that are poorly represented in genome databases.

A higher relative expression of genes in the presence of lignocellulose from the basidiomycete genera *Trametes* (a ‘white rot’ fungus) (Vasina *et al*., 2017) and *Postia* (‘brown rot’ Fungi) (Rytioja *et al*., 2014) is likely due to their specialized traits geared towards degrading lignin. Several species of *Trametes* produce lignin degrading peroxidases (Vasina *et al*., 2017) and *Postia* also produce enzymes that contribute to lignocellulose degradation (Martinez *et al*., 2009). Basidiomycete Fungi are typically less abundant in the marine environment relative to Ascomycota and Chytrid Fungi (Richards *et al*., 2012). However, despite their reduced abundance – our data suggests that these groups have potential to contribute to lignocellulose degradation in estuarine habitats.

The reduced richness in the mycobenthos after development of benthic anoxia is consistent with observations from freshwater fungi, where hypoxia and sulfide are correlated with a decreased fungal activity and abundance (Baerlocher and Boddy, 2015). However, many Fungi have the ability to degrade organic matter anaerobically via denitrification (Maeda *et al*., 2015), which is linked to the production of the trace greenhouse gas (TGG) N_2_O (Wankel *et al*., 2017). Denitrifying fungi lack a nitrous oxide reductase yet contain a unique p450nor nitric oxide (NO) reductase that produces N_2_O from NO (Shoun *et al*., 2012) – and denitrifying fungi are commonly observed in low‐oxygen marine habitats (Cathrine and Raghukumar, 2008; Jebaraj and Raghukumar, 2009; Stief *et al*., 2014). The persistence of the mycobenthos under benthic anoxia suggests that some representatives were metabolizing via denitrification. Indeed, the capability for denitrification is widespread in the genus *Aspergillus* (Stief *et al*., 2014; Maeda *et al*., 2015), which expressed a relatively high number of genes under 25% O_2_ a.s. (Fig. 6B). Because they lack N_2_O reductase, p450nor activity by denitrifying fungi results in increased production of the TGG N_2_O in coastal sediments (Wankel *et al*., 2017). Thus, the changing community structure of mycobenthos in response to organic matter inputs and benthic anoxia could also affect N_2_O fluxes from coastal ecosystems.

The increase in gene expression from Flavobacteria in the presence of lignocellulose (Fig. 6A), together with the significantly increased growth of total bacteria under 25% O_2_ a.s. relative to the control (*t*‐test: *P* = 0.01; Fig. 7A), indicates that the Flavobacteria were a major group of bacteria that were able to rapidly utilize the lignocellulose. This is consistent with a stable isotope probing study that confirmed flavobacteria are important contributors to lignocellulose cycling in salt marshes (Darjany *et al*., 2014). Even under 5% O_2_ a.s., the bacterial community was more abundant in the presence of the lignocellulose extract relative to the control, albeit with weaker statistical significance (*t*‐test: *P* = 0.05; Fig. 7A). This indicates that increased organic matter helped to sustain the size of the bacterial community under hypoxic conditions, which was due in part to the greater activity of Flavobacteria under low oxygen. Flavobacteria are one of the fastest responders to phytoplankton blooms in the North Sea where they form a bacterioplankton bloom growing quickly on the scale of days and exhibit a slower death phase over several weeks that correlates with the abundance of phytoplankton (Teeling *et al*., 2012). A similar, albeit much faster (e.g. on the scale of hours) mode of ‘boom and bust’ growth (Lennon and Jones, 2011) exhibited by bacteria in response to the lignocellulose extract shown here (Fig. 7A), where Flavobacteria dominate in terms of gene expression (Fig. 6A) and 16S rRNA genes (Fig. 6B), indicates that some Flavobacteria may act as r‐strategists in estuarine sediments.

After the development of benthic anoxia, the increased growth of nitrate reducing *Nitratireductor* and sulfate reducing *Desulfovibrio* and *Desulfosarcina* in >1 week incubations with lignocellulose, relative to the control (Fig. 7B), suggests that they were also important degraders of the added lignocellulose extract under anoxic conditions. The role of sulfate reducing bacteria (SRB) in organic matter remineralization marine sediments is well known (Jørgensen, 1982), and proceeds often through the utilization of low molecular weight volatile fatty acids (Muller *et al*., 2018). Thus, the increased growth of SRB in the samples that received lignocellulose is likely due to their utilization of low molecular weight volatiles produced during the degradation of the lignocellulose by the greater microbial community. Our results indicate that in estuarine sediments lignocellulose is degraded by consortia of Fungi, Labyrinthulomycetes, Flavobacteria, denitrifier bacteria and SRB, that occurs over a range of redox conditions that can develop rapidly (within hours). These findings support the hypothesis that organic matter in marine sediments is degraded via complex microbial consortia that includes Fungi (Orsi, 2018). The mycobenthos should be thus considered as important organisms involved in the degradation of lignocellulose at the land‐sea interface, influencing the storage of organic carbon deposited in coastal ecosystems

## Experimental procedures

### *Study site*

Surface sediment samples were collected in July 2016 from a 1 m water depth from Sage Lot pond, a coastal lagoon connected as a subestuary to Waquoit Bay (Cape Cod, MA). Sage Lot pond is a small (surface area 0.17 km^2^) shallow (ca. 2 m maximum depth) lagoon surrounded by dense vegetation including salt marshes and seagrasses (Wang *et al*., 2016), with macrophyte biomass totaling 9 (+/−1.2) mg cm^2^ (Valiela *et al*., 1992). The mouth of the Sage Lot pond has a mean annual phytoplankton chlorophyll concentration of 3.9 (+/−1.2) mg L^−1^, which can increase to 90 mg L^−1^ in the case of increased nitrogen inputs (Valiela *et al*., 1992). These eutrophic conditions lead frequent benthic anoxic events (Valiela *et al*., 1992) and a high sedimentation rate of 10 mm yr^−1^ where the sediments are composed of fine grained organic rich sandy mud (Maio *et al*., 2016). The sediments have a relatively high carbon content of 5.93% (+/−0.37) with a C:N ratio of 8.9 (+/−0.5) (Foster and Fulweiler, 2014). The water content of the sampled sediments was determined to be 15% (+/−1) of the sediment dry weight.

### *Experimental setup*

Surface sediment samples of 2 g fine grained organic rich sandy mud were overlaid with 2 ml of the natural estuarine bottom water and were added to 20 ml sterile glass vials containing sterile oxygen sensor spots (PreSens Precision Sensing) with ca. 15 ml of oxygenated headspace. The oxygen sensor spot was positioned at the sediment – seawater interface to measure benthic O_2_ concentrations, and additional sensor spots were placed in the headspace of two flasks to measure gaseous O_2_ levels throughout the incubation. Lignocellulose was extracted from wheat stems as described previously (Crawford and Crawford, 1976), pulverized into powder and added to the sediment at a concentration of 5 mg per g sediment in triplicate. This concentration of organic matter is realistic for the sampled lagoon where average macrophyte biomass is 9 (+/−1.2) mg cm^2^ (Valiela *et al*., 1992) and carbon content in the upper meter of sediment is roughly 6% (Foster and Fulweiler, 2014). Separate vials that did not receive lignocellulose were also set up as controls. Vials were crimp sealed with 15 ml of headspace using sterile rubber stoppers and incubated in the dark at room temperature without shaking. Whole vials were taken (sacrificed) in triplicate for ITS1 and 16S rRNA gene sequencing at T0, 25%, 5% and 0% benthic O_2_ concentrations (a.s.) for treatments that received, or did not receive (control), lignocellulose. Samples for metatranscriptomics were taken only from the 25% benthic O_2_ concentrations. Sacrificing whole vials at each time point avoided the opening and re‐crimping of vials, which would have altered the O_2_ concentrations in the flasks. O_2_ was continuously measured several times throughout the day with the Fibox 4 using oxygen sensor spots (PreSens Precision Sensing). Replicates were then stored frozen (−20 °C) for downstream analyses.

### *DNA extraction*

DNA was isolated from 0.5 g sediment incubations aseptically in a sterile laminar flow hood using a sterile spatula, and extractions were carried out as described previously (Pichler *et al*., 2018). Purification of DNA extracts was carried out with the PowerClean Pro DNA Clean‐up Kit (MO BIO Laboratories) and DNA was quantified with the Qubit dsDNA HS Assay kit (Thermo Fisher Scientific) according to manufacturer's instructions. For preparation of fungal mock community ITS1 sequencing libraries, 13 different freeze‐dried cultures (Table 1) were purchased from the Leibniz‐Institute DSMZ‐German Collection of Microorganisms and Cell Cultures, rehydrated in lysogeny broth (LB) following the manufacturer's instructions and grown on agar plates (yeast extract‐peptone‐dextrose [YPD] broth) for 3–7 days at room temperature. DNA was extracted using a previously described protocol (Pichler *et al*., 2018). Three technical replicates of the mock community were created to assess variation in Illumina sequencing fidelity on the MiniSeq platform across two different sequencing runs. DNA from laboratory dust and extraction blanks was extracted and purified in order to identify and remove any contaminant sequences introduced during the laboratory processing of the samples.

### *ITS1 sequencing*

The fungal ITS1 region from each sample was amplified with the primer pair ITS1‐F/ITS2 that contain in addition to the primer sequence the Illumina adaptors and a unique barcode sequence (Walters *et al*., 2016). For each sample, three technical replicates were prepared with the epMotion 5070 robotic pipetting system (Eppendorf). Reactions were set up in 20 μl volumes, containing 10 μl Sso Advanced Universal SYBR Green Supermix (Bio‐Rad Laboratories), 6.8 μl Nuclease‐free H_2_O, 0.4 μl primers (10 μM, biomers.net), 0.4 μl MgCl_2_ and 2 μl template. Cycling conditions comprised 95 °C for 2 min, followed by 40 cycles of 95 °C for 15 s, 51 °C for 30 s and 72 °C for 1 min 30 s. ITS1 amplicons were purified with QIAquick Gel Extraction Kit (Qiagen) and quantified with the Qubit dsDNA HS Assay Kit (Thermo Fisher Scientific). Purified PCR amplicons containing unique barcodes from each sample were diluted to 1 nM solutions and pooled. Library preparation was carried out according to the MiniSeq System Denature and Dilute Libraries Guide from Illumina. Sequencing was performed on the Illumina MiniSeq as described previously for 16S rRNA genes (Pichler *et al*., 2018), containing 350 μl library (1.8 pM), 150 μl denatured genome library (1.8 pM) and 15 μl denatured PhiX control, and the Illumina MiniSeq Mid Output Kit (300‐cycles).

### *qPCR*

Bacterial 16S rRNA gene V4 hypervariable region was performed with primer pair 515F/806R (Walters *et al*., 2016) using the qPCR protocol described previously (Coskun *et al*., 2018). In brief, qPCR was carried out in 20 μl solutions containing 10.4 μl SsoAdvanced SYBR green PCR buffer (Bio‐Rad, Hercules, CA, USA), 0.4 μl of 10 mM primer, 6.8 μl of nuclease‐free water and 2 μl of the DNA template. Three technical replicates were prepared with the epMotion 5070 robotic pipetting system (Eppendorf), with a technical variation of <5%. All reactions were performed with a two‐step protocol in a CFX Connect real‐time PCR system (Bio‐Rad, Hercules, CA, USA), including an enzyme activation step at 95 °C for 3 min, followed by 40 cycles of denaturation at 95 °C for 15 s and then annealing at 55 °C for 30 s. qPCR standards consisted of 10‐fold dilution series of the genes of interest that were PCR amplified from the sample using the same primers. Prior to the creation of the dilution series, the amplified standard was gel extracted and quantified with a Qubit instrument. The reaction efficiencies in all qPCR assays were between 90% and 110%, with an r^2^ of 0.98. Gene copies were normalized to the wet weight of the sediment. Sequencing of the amplicons on the Illumina MiniSeq was performed as described previously (Pichler *et al*., 2018) through the LMU Munich GeoBio Center.

### *Bioinformatic analysis*

For both the ITS1 and 16S rRNA genes, we performed demultiplexing and base calling using bcl2fastq Conversion Software v2.18 (Illumina) and USEARCH (Edgar, 2013) as described previously (Pichler *et al*., 2018). ITS1 reads were clustered into OTUs sharing 97% sequence identity and taxonomy was assigned with an identity threshold of 90% against the UNITE ITS database (Koljalg *et al*., 2013) using BLASTn. For 16S rRNA genes taxonomic assignments were generated by QIIME, version 1.9.1 (Caporaso *et al*., 2010), using the implemented BLAST method against the SILVA rRNA gene database, release 128 (Quast *et al*., 2013). We removed all OTUs containing <3 sequences and which had no BLASTn hit. All OTUs that were found in the dust and extraction blank (contamination) samples were also removed from the dataset. Reads passing this quality control were then normalized by percentage of total sequencing depth per sample. In the ITS1 mock community, false positive OTUs were identified as those OTUs with a BLASTn hit to organisms that were not in the original mock community.

FUNGuild (Nguyen *et al*., 2016) was used to predict the trophic status of the mycobenthos taxa. FUNGuild uses a carefully hand curated database drawing upon physiological literature, to group fungal organisms broadly into either saprotrophic, pathotrophic, symbiotrophic groups (with some groups exhibiting more than one lifestyle) according to Tedersoo *et al* (Tedersoo *et al*., 2014). The groups are defined as follows: (i) saprotrophs receive nutrients by breaking down dead host cells; (ii) pathotrophs receive nutrients by harming living host cells; (iii) symbiotrophs receive nutrients by exchanging resources with living host cells. These broadly defined trophic modes reflect the major feeding habits of fungi (Tedersoo *et al*., 2014; Treseder and Lennon, 2015, Nguyen *et al*., 2016).

### *Metatranscriptomics*

Total RNA was extracted from 0.7 g of incubation slurry from the lignocellulose treatment, and control treatment, at 25% O_2_ a.s. We applied the FastRNA Pro Soil‐Direct Kit (MP Biomedicals) following the manufacturer's instructions with final elution of templates in 30 μl PCR‐grade water (Roche). RNA extracts were quantified using the QuBit RNA HS Assay Kit (Thermo Fisher Scientific), with corresponding concentrations of 6.2 and 5.3 ng × μL^−1^ for each sample. DNAse treatment, synthesis of complementary DNA and library construction were obtained from 10 μl of RNA templates by processing the Trio RNA‐Seq kit protocol (NuGEN Technologies). Libraries were quantified on an Agilent 2100 Bioanalyzer System, using the High Sensitivity DNA reagents and DNA chips (Agilent Genomics). The libraries constructed using specific (different) barcodes, pooled at 1 nM and sequenced in two separate sequencing runs with a paired‐end 300 mid output kit on the Illumina MiniSeq. A total of 10.5 million sequences were obtained after Illumina sequencing, which could be assembled *de novo* into 19 259 contigs. Quality control, *de novo* assembly, read mapping, and open reading frame (ORFs) searches were performed as described previously (Orsi *et al*., 2018). Only contigs with an average coverage >5 were selected for ORF searches, and downstream analysis. ORFs were then searched for similarity using BLASTp against a database containing predicted proteins from all fungal, bacterial and archaeal genomes in the NCBI RefSeq database – updated with predicted proteins from >700 transcriptomes recently sequenced from marine eukaryotic microbes, including several fungal‐analogue lineages including those from the Labyrinthulomycetes (Keeling *et al*., 2014). Cutoff for assigning hits to specific mycoplankton taxa were a minimum bit score of 50, minimum amino acid similarity of 60 and an alignment length of 50 residues. All sequence data is publicly accessible in NCBI through BioProject number PRJNA473897.

## Supporting information


**Table S1.** List of all fungal taxa detected in the contamination controls sequenced from DNA extraction blanks and dust samples collected from the lab.
